# Overexpression of mtDNA-associated AtWhy2 compromises mitochondrial function

**DOI:** 10.1186/1471-2229-8-42

**Published:** 2008-04-18

**Authors:** Alexandre Maréchal, Jean-Sébastien Parent, Mohammed Sabar, Félix Véronneau-Lafortune, Charbel Abou-Rached, Normand Brisson

**Affiliations:** 1 Department of Biochemistry, Université de Montréal, 2900 Édouard-Montpetit, Montréal, Québec, H3C 3J7, Canada

## Abstract

**Background:**

StWhy1, a member of the plant-specific Whirly single-stranded DNA-binding protein family, was first characterized as a transcription factor involved in the activation of the nuclear *PR-10a *gene following defense-related stress in potato. In *Arabidopsis thaliana*, Whirlies have recently been shown to be primarily localized in organelles. Two representatives of the family, AtWhy1 and AtWhy3 are imported into plastids while AtWhy2 localizes to mitochondria. Their function in organelles is currently unknown.

**Results:**

To understand the role of mitochondrial Whirlies in higher plants, we produced *A. thaliana *lines with altered expression of the *atwhy2 *gene. Organellar DNA immunoprecipitation experiments demonstrated that AtWhy2 binds to mitochondrial DNA. Overexpression of *atwhy2 *in plants perturbs mitochondrial function by causing a diminution in transcript levels and mtDNA content which translates into a low activity level of respiratory chain complexes containing mtDNA-encoded subunits. This lowered activity of mitochondria yielded plants that were reduced in size and had distorted leaves that exhibited accelerated senescence. Overexpression of *atwhy2 *also led to early accumulation of senescence marker transcripts in mature leaves. Inactivation of the *atwhy2 *gene did not affect plant development and had no detectable effect on mitochondrial morphology, activity of respiratory chain complexes, transcription or the amount of mtDNA present. This lack of phenotype upon abrogation of *atwhy2 *expression suggests the presence of functional homologues of the Whirlies or the activation of compensating mechanisms in mitochondria.

**Conclusion:**

AtWhy2 is associated with mtDNA and its overexpression results in the production of dysfunctional mitochondria. This report constitutes the first evidence of a function for the Whirlies in organelles. We propose that they could play a role in the regulation of the gene expression machinery of organelles.

## Background

Plant cells comprise three organelles (nucleus, plastids and mitochondria) that possess and maintain genetic information. Coordination of gene expression in these organelles is critical for plant development and survival [1,2]. Since the endosymbiosis events that resulted in the integration of plastids and mitochondria into eukaryotic cells, most of the genetic information found in the cyanobacterial and α-proteobacterial ancestors has been transferred to the nucleus. Nevertheless, remnants of the original genomes are still found in organelles. In Arabidopsis, the mitochondrial genome contains coding sequences for approximately 87 genes encoding mainly components of the translational apparatus and of the electron transport chain [3]. Since no protein involved in general DNA metabolism is present in the mitochondrial genome of Arabidopsis, gene expression in this organelle is under nuclear control. A consequence of this is that extensive anterograde (nucleus to organelle) and retrograde (organelle to nucleus) signalling is required for co-regulation of nuclear and organellar genes that encode proteins working cooperatively in organelles as well as for the general homeostasis of mitochondria.

Whirlies form a small family of single-stranded DNA (ssDNA) binding proteins found mainly in the plant kingdom. StWhy1, the prototypical Whirly from *Solanum tuberosum*, has been characterized as a transcriptional activator of the pathogenesis-related gene *PR-10a *following elicitation or wounding of potato tubers [4-6]. Following stress, it was shown to bind with high affinity to a single-stranded form of an inverted-repeat-containing region located in the promoter of *PR-10a *called the elicitor response element (ERE) both *in vitro *and *in vivo *[6]. Analysis of the crystal structure of StWhy1 revealed that *in vivo *Whirlies adopt a tetrameric form. Each protomer consists of two antiparallel β sheets packed perpendicularly against each other forming blade-like extensions which protrude out of an α-helical core that allows formation of a stable tetramer. The surface formed by these "blades" was proposed to form the Whirly ssDNA-binding domain [7]. In accordance with the role of StWhy1 in *S. tuberosum*, the Arabidopsis homolog AtWhy1 was shown to be required for both full basal and specific disease resistance responses to the obligate biotroph *Peronospora parasitica *[6].

Based on analysis of the primary sequence of Whirly proteins from a variety of flowering plants, we predicted that they could localize to organelles [5]. Recently, this was confirmed for the *Arabidopsis thaliana *Whirly representatives. Two of those Whirlies, AtWhy1 [TAIR:At1g14410] and AtWhy3 [TAIR:At2g02740] are imported in plastids whereas AtWhy2 [TAIR:At1g71260] is targeted to the mitochondria ([8] and our unpublished data). Remarkably, all flowering plants, when sufficient sequence information is available, contain at least two Whirly representatives, one predicted to be plastid-localized while the other is expected to be in mitochondria. In a recent turn of events, another nuclear function has been proposed for the Whirlies as AtWhy1 was shown to be involved in telomere length homeostasis [9]. Although dual-localization of Whirlies to nucleus and organelles in the same cell remains to be shown, it is possible that under certain circumstances, such as specific stresses or developmental cues, Whirlies could shuttle between cell compartments, thus representing good candidates as mediators of antero/retrograde signalling. In a first step towards a better understanding of the relationship between the nuclear functions of the Whirlies and their primary localization to organelles, we decided to elucidate the functions of the Whirlies in mitochondria.

## Results

### Overexpression of AtWhy2 perturbs mitochondrial function

**Figure 1:**
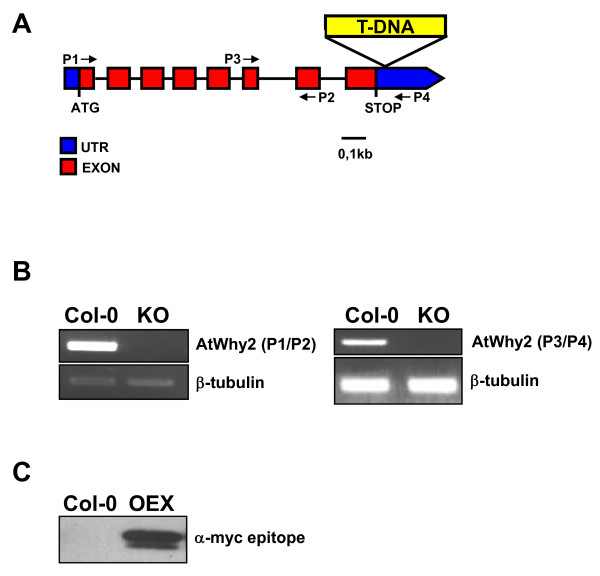
**Production of plants with altered expression of *atwhy2***. **A**. Physical map of the *atwhy2 *(AT1G71260) gene. The position of the T-DNA insertion in the KO line is indicated. The small arrows symbolize the primers used to amplify *atwhy2 *mRNA by RT-PCR. **B**. Molecular analysis of plants homozygous for the disrupted *atwhy2 *allele. RT-PCR was performed on Col-0 and KO total RNA samples using two sets of primers (P1/P2 and P3/P4). Semi-quantitative conditions were used and primers for tubulin amplification were used as a control. **C**. Levels of the AtWhy2-myc fusion protein in OEX and wild-type (Col-0) plants were monitored by Western blot using a monoclonal antibody against the c-myc epitope.

**Figure 2:**
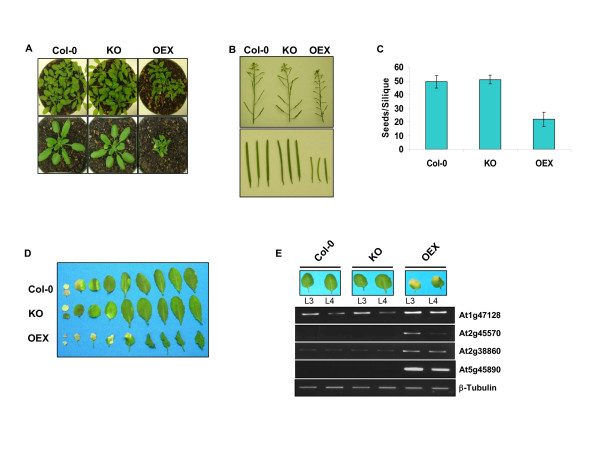
**Phenotypic characterization of plants with altered expression of *atwhy2***. **A**. Four week old plants of the indicated genotypes grown in soil were photographed. **B**. Representative inflorescences and individual siliques taken from six week old plants of the indicated genotypes were photographed. **C**. Twenty individual mature siliques from the indicated genotypes were dissected and their average seed content was calculated. **D**. Equivalent leaves were taken from 6 week old plants and photographed. Leaves are ordered by age from left to right. **E**. Early accumulation of senescence marker transcripts in mature leaves of OEX plants. RT-PCR was performed on Col-0, KO and OEX RNA samples taken from the 3^rd ^(L3) and 4^th ^(L4) leaves of 5 week old plants using oligonucleotides designed to amplify specifically the following genes: At1g47128: Cystein protease RD21A, At2g45570: YLS6, Cell-death-associated cytochrome, At2g38860: YLS5, Protease I and At5g45890: SAG12, Cystein protease. Semi-quantitative conditions and primers for β-tubulin amplification were used to ensure adequate loading for all samples.

To investigate the role of mitochondrial Whirlies, we produced plants with altered expression of the *atwhy2 *(At1g71260) gene. Homozygous plants carrying a T-DNA insertion in the 3' untranslated region and completely devoid of *atwhy2 *expression were obtained (Figure 1A and 1B). In addition, plants constitutively overexpressing a myc-tagged version of AtWhy2 under the control of the CaMV 35S promoter were produced (Figure 1C). While the knock-out plants (KO) showed no visible phenotype (Figure 2A), plants overexpressing *atwhy2 *(OEX) were smaller and produced shorter siliques containing about half the amount of seeds found in wild-type plants (Figure 2B and 2C). Interestingly, OEX plants also developed dark green distorted leaves and their mature leaves exhibited signs of early senescence when compared to wild-type (Col-0) or KO plants (Figure 2A and 2D). To document this accelerated cell death, we monitored the expression levels of a number of previously described senescence-associated genes (SAGs) in the third and fourth leaves of 5 week old plants from each genotype using RT-PCR (Figure 2E). These genes have all been described as molecular markers of leaf senescence because their abundance is significantly increased during this genetically programmed phenomenon [10,11]. The mRNAs of all tested SAGs were clearly more abundant in OEX compared to Col-0 and KO plants, thereby confirming that the early yellowing of leaves is an indication that a senescent state is reached more rapidly in leaves of plants overexpressing *atwhy2*. To ascertain that the observed phenotypes were not due to a non-specific effect of the overexpression of a mitochondria-targeted myc-tag, we produced transgenic plants constitutively expressing an untagged version of AtWhy2 and observed the same phenotypes (data not shown).

**Figure 3:**
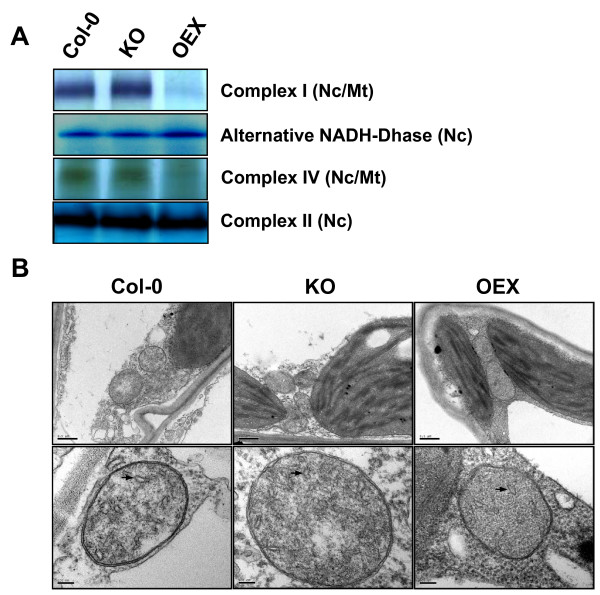
**Mitochondrial perturbation due to overproduction of AtWhy2**. **A**. Activity of mitochondrial respiratory chain complexes in plants with altered expression of *atwhy2*. BN-PAGE was used to separate membrane protein complexes from crude mitochondrial fraction taken from plants of the indicated genotypes. Activity of the different complexes was evaluated by in-gel enzymatic assays using equivalent protein amounts for all plants. **B**. Mitochondria ultrastructure was evaluated using transmission electron microscopy. In the upper panel, representative mitochondria from the indicated genotypes were photographed at 25000 × magnification. The bar represents 0.5 μm. In the lower panel, organelles were observed at 100000 × magnification. The bar represents 100 nm. Black arrows point to invaginations of the inner membrane (cristae).

Recent reports have highlighted the role of mitochondria in regulation of senescence in numerous organisms including yeast, flowering plants and mammals [12-15]. As it has been demonstrated that AtWhy2 is imported into mitochondria *in vivo*, these observations prompted us to verify whether mitochondria in plants with altered *atwhy2 *expression were still functional. In order to monitor the activity of the mitochondrial respiratory chain complexes, we used blue-native polyacrylamide gel electrophoresis (BN-PAGE) coupled to in-gel histochemical staining of enzymatic activities [16]. Using this procedure, we were able to evaluate the individual activities of NADH dehydrogenases, succinate dehydrogenase (Complex II) and cytochrome C oxidase (Complex IV). No differences in activity for all the observed complexes could be found between Col-0 and KO plants (Figure 3A). In contrast, OEX plants exhibited strong deficiencies in Complexes I and IV while their alternative NADH dehydrogenase and Complex II remained as competent as those found in wild-type and KO. Interestingly, only complexes containing subunits encoded by the mitochondrial genome were affected in the OEX plants. Complexes composed exclusively of polypeptides encoded in the nuclear genome were intact. These observations prompted us to use electron microscopy to monitor the quantity and ultrastructure of organelles present in the various lines. All plants contained approximately the same number of mitochondria that were of similar size (Figure 3B upper panel). At higher magnification, mitochondria from OEX plants exhibited a simpler structure than those in Col-0 and KO plants. In general cristae were slightly less abundant in OEX plants (Figure 3B lower panel (black arrows)). The invaginations of the inner membrane were counted on 10 mitochondrial sections of similar size for each of the genotypes. Cristae density averages for all observed sections (in cristae/μm^2^) were 24.2 ± 7.9, 23.3 ± 10.3 and 17.1 ± 7.1 for Col-0, KO and OEX plants respectively. Altogether, these results indicate that mitochondrial function is compromised upon overexpression of *atwhy2*.

### General downregulation of mitochondrial gene expression and mtDNA levels in plants overproducing AtWhy2

The ssDNA-binding capacity of the Whirlies could be an important regulator of gene expression in organelles. Since StWhy1 has been shown to act as a transcriptional activator for the *PR-10a *nuclear gene in tubers following elicitation, it is plausible that AtWhy2 could take part in the regulation of transcription in mitochondria [4,6]. This eventual function was investigated by monitoring mitochondrial gene expression in plants with altered *atwhy2 *content using RNA gel blots.

**Figure 4:**
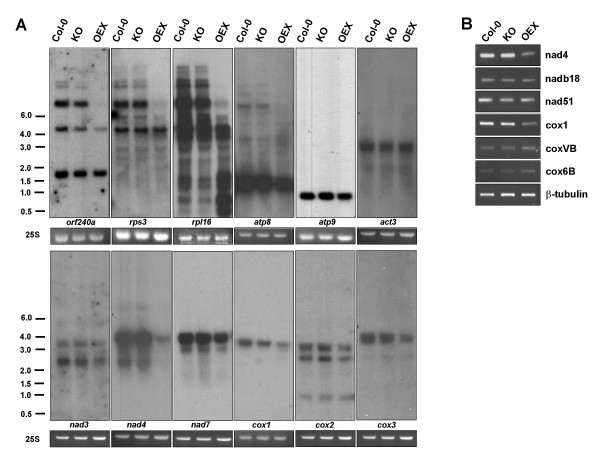
**Gene expression of mtDNA- and nuclear-encoded genes coding for mitochondrial proteins**. **A**. RNA gel blot analysis of mitochondrial genes. 10 μg of total RNA extracted from 100 mg of mature leaf tissue were separated on a 1.2% agarose gel and blotted onto a nylon membrane. PCR-amplified probes were used for the detection of the indicated genes. Ethidium bromide-stained 25S rRNA provides the RNA loading control. **B**. RT-PCR was performed on Col-0, KO and OEX total RNA extracted from leaves of 4 week old plants. Amplified genes are indicated. Semi-quantitative conditions and primers for β-tubulin amplification were used to ensure adequate loading for all samples.

Since respiratory chain complexes I and IV function is compromised in OEX plants, we evaluated the expression levels of three subunits from each of these complexes that are encoded by the mitochondrial genome. As shown in Figure 4A, steady-state RNA levels for *nad3*, *nad4*, *nad7 *and for *cox1*, *cox2 *and *cox3 *were all significantly reduced in OEX plants compared to Col-0 and KO plants. No change could be observed between Col-0 and KO plants for the steady state RNA levels detected with all probes. This is in agreement with the similar activity observed for the respective supercomplexes (Figure 3A). Similar results were obtained for mitochondrial genes *atp8, atp9, orf240a, rps3 *and *rpl16*. Upon closer examination we observed that the smallest RNA forms, presumably representing the mature translated RNA, are usually less affected than the larger forms, which may represent the primary transcripts. Surprisingly, for the *rpl16 *probe, the smallest RNA products were more abundant in OEX compared to KO and wild-type plants. We propose that these differences between the abundance of RNAs of different sizes could be due to post-transcriptional stabilization compensating for the reduced production of the large primary transcripts.

AtWhy2 has never been detected in the nucleus and is consistently described as a mitochondrial protein ([8] and our unpublished data). However it still remains possible that at least part of the deficiency in mitochondrial complexes observed in OEX plants could be due to a defect in the expression of nuclear genome-encoded respiratory chain subunits or to a general defect in RNA metabolism. To verify this, we first tested nuclear 25S rRNA levels by ethidium bromide staining. Figure 4A shows that the amount of 25S rRNA did not vary in the three types of plants. We then measured the levels of *act3 *mRNA, coding for actin and showed that these levels remained unchanged in all the plants (Figure 4A). We also evaluated the expression of two different nuclear genes encoding subunits of both the NADH dehydrogenase (Complex I) and of the cytochrome C oxydase (Complex IV) using semi-quantitative RT-PCR. No differences in expression levels could be observed among Col-0, KO and OEX plants for the *nadb18 *[TAIR:At2g02050], *nad51 *[TAIR:At5g08530], *coxVb *[TAIR:At1g80230] and *cox6b *[TAIR:At1g22450] genes (Figure 4B). Oligonucleotides designed to amplify a β-tubulin cDNA were used as a loading control. Transcript levels for *nad4 *and *cox1 *cDNA, which are encoded in the mitochondrial genome, were reduced in OEX plants (Figure 4B), as also shown by RNA gel blot (Figure 4A). Altogether, the data presented here underlines that mitochondrial RNA accumulation is specifically affected by overexpression of AtWhy2. These observations suggest that AtWhy2 might affect maturation of RNA in mitochondria. Alternatively, it could affect transcription or lead to a reduction in the amount of mitochondrial DNA (mtDNA) which in turn could result in a drop in transcript accumulation for all the genes encoded.

**Figure 5:**
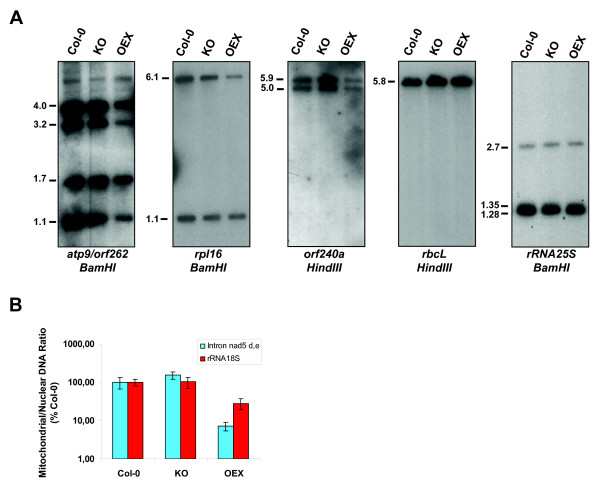
**Mitochondrial, plastidial and nuclear DNA content in plants with altered expression of *atwhy2***. **A**. DNA gel blot analysis of mitochondrial, plastidial and nuclear genes. 10 μg of total DNA digested with the indicated restriction enzymes were separated on a 1% agarose gel, blotted onto a nylon membrane and hybridized with probes representing the indicated genes. Numbers on the left indicate the molecular weight of the observed bands in kilobasepairs (Kb) as predicted by restriction mapping of mtDNA. **B**. Ratio of mitochondrial to nuclear genomes was evaluated by Quantitative Real-Time PCR using 2 different mitochondrial gene regions and a single nuclear genome region. The mt/nuclear DNA ratio of Col-0 plants was arbitrarily fixed as 100%.

To assess this latter possibility, we monitored the level of mtDNA in the plants. Probes directed against the *atp9*, *rpl16 *and *orf240a *coding sequences of the mitochondrial genome were used to evaluate the amount of mtDNA present in Col-0, KO and OEX plants by Southern blot. All mitochondrial DNA regions detected by these probes were found to be reduced in abundance in plants overexpressing AtWhy2 (Figure 5A). No difference in the amount of mtDNA could be observed between wild-type and KO individuals. The *rpl16 *probe detected a band of 6.1 kb following digestion of total DNA with *BamHI*. The intensity of this band, which corresponds to the coding region of *rpl16 *in the mitochondrial genome, is significantly lower in OEX plants. This probe also detected a nuclear sequence found on chromosome II in the form of a band of 1.1 kb that remained unaffected in plants overexpressing *atwhy2*, indicating that the amount of nuclear DNA in OEX plants is similar to that of KO and Col-0 plants. To confirm that mtDNA is specifically affected by the overexpression of AtWhy2, we also used probes directed against the plastidial gene *rbcL *and the nuclear gene encoding the 25S rRNA. We found that both of these sequences are present in equal amounts in Col-0, KO and OEX plants. Thus the observed reduction in the expression level of mitochondrial genes correlates with a reduced level of mtDNA. The reduced amount of mtDNA in the OEX plants was confirmed by quantitative PCR (qPCR). Primers were designed to amplify unique regions of the mitochondrial and nuclear genomes and DNA ratios of the two genomes were compared. As with the regions quantified by Southern blotting, no difference between mt/nuclear DNA ratios could be highlighted by comparing Col-0 and KO DNA samples (Figure 5B). However and in agreement with the DNA gel blot results, both OEX mitochondrial regions gave significantly reduced mt/nuclear DNA ratios (7.1% of wild-type ratio for the intron between exons d and e of *nad5 *and 28.2% of wild-type ratio for a portion of the *rRNA 18S *gene).

It has been shown that changes in the mitochondrial recombination regulation system of higher plants can result in the accumulation of normally substoichiometric DNA molecules (sublimons) [17-19]. As Whirlies bind to ssDNA, a role in DNA recombination cannot be excluded. A particularly active site of recombination in the mitochondrial genome of *A. thaliana *is located around the *atp9 *gene. This region contains numerous small repeats that are able to recombine to produce new DNA molecules. To investigate a potential role of AtWhy2 as a regulator of mtDNA recombination, we digested total DNA from the three lines with the BamHI restriction enzyme and observed the various bands detected with a probe encompassing the whole *atp9 *gene and the 5' portion of *orf262*. The expected 4 bands of 1.1, 1.7, 3.2 and 4.0 kb were present in all of the genotypes though in reduced amount in OEX (see [17] for identification of the individual bands) (Figure 5A). AtOSB1, a mitochondria-localized member of the Organellar Single-stranded DNA Binding protein family, RecA3 and MSH1, a MutS family homolog (formerly CHM1) have been implicated in the regulation of mitochondria recombinational activity [17-20]. When these genes are knocked-out, they produce a phenotype similar to that observed upon overexpression of AtWhy2 (distorted leaves). Absence of these genes also results in the accumulation of aberrant recombination products that are visible by Southern blotting when using probes directed against the *atp9 *region. In contrast to these mutants, no new band appeared in the AtWhy2 KO or OEX plants. Additionally, with two other probes directed against *rpl16 *and *orf240a*, we could not observe an accumulation of putative aberrant recombination products, suggesting that AtWhy2 is not involved in the regulation of recombination in mitochondria. However, although we could not detect changes around the *atp9*,*rpl16 *and *orf240a *regions, we examined only 8.2% of the Arabidopsis mitochondrial genome (28.2 kb out of approximately 350 kb). We therefore cannot eliminate the possibility that recombination might have occurred among other repeats present in mtDNA of OEX plants. Interestingly, segregating heterozygous OEX plants produce 1/4 of wild-type plants that behave as the original Col-0 ecotype (data not shown). This suggests that overproduction of AtWhy2 does not affect the mitochondrial genome in a permanent manner and that an increased amount of this protein is necessary to generate and maintain the observed mitochondrial defects.

### AtWhy2 is associated with mitochondrial DNA

**Figure 6:**
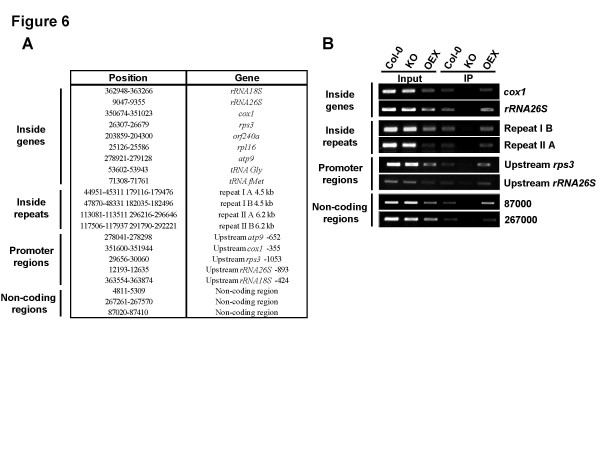
**AtWhy2 is associated with mitochondrial DNA**. **A**. Position of the amplified regions on the mitochondrial genome. **B**. PCR amplification of mtDNA regions following immunoprecipitation on crude mitochondrial extracts of the indicated genotypes. Inputs represent 20% of the total DNA used in the immunoprecipitation. Representative regions are shown here, other amplified regions gave similar results showing specific interaction with AtWhy2.

In *Chenopodium album *mitochondrial DNA, long and abundant stretches of single-stranded DNA are present and distributed all over the genome [21]. Presence of ssDNA has also been reported in the mitochondria of cultured tobacco cells [22]. Therefore, in those species, some of these regions could be bound by mitochondria-targeted Whirlies to control a yet-to-be-defined process in plants. To determine if and which DNA regions are bound by AtWhy2 in Arabidopsis, we immunoprecipitated AtWhy2 and detected DNA bound to it by PCR amplification (Figure 6). Oligonucleotides were designed to amplify four types of DNA regions based on their biological significance: in the vicinity of promoter regions [23], inside genes, inside isolated regions that are devoid of any coding sequence and inside the two large repeated regions of the mitochondrial genome (Figure 6A).

In these experiments, we predict that a region that is specifically pulled-down using a polyclonal anti-AtWhy2 antibody should amplify up to a certain level for Col-0 plants and should amplify to equal or higher levels in the OEX. In KO plants, PCR products should be absent or lower than in both OEX and Col-0 plants (mtDNA being especially abundant in plant cells, some non-specific immunoprecipitation may occur). Figure 5B (Input), indicates that for all amplified regions, the amount of mtDNA was lower in the OEX than in the Col-0 and KO plants, confirming our previous observations about the reduced amount of mtDNA in this mutant line. After immunoprecipitation, all tested DNA regions were pulled-down in the Col-0 and OEX plants but not, or to a much lesser extent in the KO plants, indicating that AtWhy2 constitutively interacts with these different regions in the mitochondrial genome (Figure 5B, IP). Interestingly, even though the input amount of mtDNA in OEX plants was lower than in Col-0 or KO, we were usually able to immunoprecipitate equivalent or higher amounts of DNA from these extracts compared to lysates from Col-0 plants, indicating that a much higher proportion of the total mtDNA was bound to AtWhy2 in overexpressing plants. Therefore, these results suggest that AtWhy2 binding to mtDNA does not discriminate between coding and non-coding sequences.

## Discussion

To narrow down the array of potential roles of mitochondrial Whirlies in higher plants, we produced *A. thaliana *lines with altered expression of the *atwhy2 *gene. Overexpression of *atwhy2 *in Arabidopsis compromised mitochondrial function by causing a reduction in transcript levels and mtDNA content. This resulted in a lowered activity of the electron transport chain complexes containing subunits encoded in the chondriome. As no significant diminution in the amount of mitochondria per leaf area was observed, the reduced amount of mtDNA in the OEX indicates that these organelles possess lower genome copy number than their counterparts in Col-0 and KO plants. Our results suggest that the modified mitochondrial activity of OEX plants may be responsible for the smaller size of these plants and the more rapid leaf senescence phenotype observed which correlates with the early induction of senescence marker genes. It has recently been reported that in yeast, a drop in mitochondrial gene expression correlates with a diminution in chronological lifespan. Moreover, an imbalance in the various components of the electron transport chain seems to be even more detrimental to yeast lifespan (reviewed in [12]). In the case of OEX plants, both of these conditions are present, as we observed a general downregulation of mtDNA-encoded gene expression coupled to an imbalance in the oxidative phosphorylation machinery. Indeed, the activity of the succinate dehydrogenase complex, which is entirely encoded in the nucleus in Arabidopsis, is unaffected in the OEX plants, while activity of the mt-encoded complexes is greatly perturbed (Figure 3A). Thus, our results suggest that similar mitochondrial dysfunction in yeast and plants are able to affect the effective lifespan of cells.

The plants with altered mitochondrial Whirly expression allowed us to determine that AtWhy2 is constitutively associated with mtDNA, most probably through a direct interaction of the Whirly domain with abundant ssDNA found in mitochondrial nucleoids. Binding sites seem to occur along the length of the mitochondrial genome since we were unable to detect a region where Whirlies are not bound to mtDNA. Interestingly, plastidial Whirlies have been isolated within Arabidopsis and mustard purified fractions containing transcriptionally active chromosomes, suggesting that they also interact with nucleoid DNA in chloroplasts [24]. Because a higher proportion of mtDNA is bound by AtWhy2 in OEX plants (Figure 5B), it is possible that an overabundance of mitochondrial Whirlies could modify gene expression in mitochondria by rendering nucleoid DNA unavailable for binding by components of the transcription and/or replication machinery, thereby diminishing the steady-state RNA levels and the amount of mtDNA produced.

Since their discovery, members of the Whirly family have been purified independently using ssDNA affinity chromatography by our and other groups [4,25,26]. While our lab used a single-stranded form of the inverted-repeat-containing elicitor response element as a bait [4], Vermel and colleagues used an affinity column exhibiting random bovine ssDNA sequences [25] and yet another group used an hexameric repeat of Arabidopsis telomere ends [26]. The fact that Whirlies are able to bind with high affinity to all these different DNAs indicates weak sequence specificity at least *in vitro*. Our mtDNA immunoprecipitation results suggest that this low sequence specificity is also true for mitochondrial Whirlies *in vivo*. As the size of DNA fragments following sonication was determined to be approximately between 350 and 1000 bp (data not shown), an alternative explanation for the obtained results could be that binding of AtWhy2 to mtDNA is specific to certain sequences that are common to the various immunoprecipitated regions.

### Putative functions of mitochondrial Whirlies

Our results suggest that AtWhy2 is involved in the regulation of mitochondrial gene expression. Whirlies are found in mitochondria and plastids from higher plants. In plastids, transcription is under the control of two types of RNA polymerases, a unique eubacterial-type plastid-encoded polymerase (PEP) and multiple phage-type nucleus-encoded polymerases (NEPs) (reviewed in [27] and [28]). These RNA-polymerases specifically regulate the transcription of different subsets of genes but can also co-regulate a portion of the plastidial genes [29]. In mitochondria of higher plants, transcription is regulated by NEPs only. These facts, coupled to the demonstrated role of StWhy1 as a transcriptional activator suggest that the Whirlies could also act as transcriptional regulators in organelles. For example, they might constitute processivity cofactors for phage-type RNA polymerases. It can be argued that since binding of AtWhy2 is also observed in non-coding regions of the mitochondrial genome, transcription might not constitute the sole function of this Whirly. However, binding to non-coding regions and to non-consensus sites has been reported for numerous classical nuclear transcription factors during genome-wide protein-DNA interaction studies (reviewed in [30]), hence a role in transcription of mtDNA-encoded genes for AtWhy2 remains possible.

In mammals, transcription is important for the replication of mitochondrial DNA, providing the primers necessary for initiation (reviewed in [31]). In higher plants however, such an interrelationship between these processes remains to be shown. The diminution in mtDNA content observed in OEX plants hints at a role for AtWhy2 in replication/maintenance of the mitochondrial genome. A distant homolog of the Whirlies, the TIF1 protein from the protist *Tetrahymena thermophyla *has been implicated in replication of DNA as well as in the faithful intergenerational transmission of genetic information and is required for activation of an intra-S-phase checkpoint triggered by DNA-damage [32-34]. Thus, mitochondrial Whirlies could potentially play multiple roles in the replication, maintenance and transcription of the chondriome.

Since overexpression of AtWhy2 differentially affects various forms of mitochondrial RNA, it is plausible that Whirlies might be implicated in the maturation of mtRNA. For example they might stabilize specific forms of RNA or facilitate processing from polycistronic immature RNA to the final translatable RNA form. Recently, the crystallographic structure of a heterotetramer of the MRP1 and MRP2 mitochondrial RNA-binding proteins from *Trypanosoma brucei *was solved. Although these two proteins share no primary sequence similarity with the Whirlies, they adopt a Whirly-like fold. These proteins are involved in RNA editing and bind to guide RNAs (gRNAs) that specify where uracil residues are added or removed in the sequence of the mitochondrial pre-mRNA [35]. We tested whether the Whirlies could have a role in editing by cloning and sequencing *nad7 *and *ccb206 *cDNAs. These RNA are normally edited at 28 (*nad7*) and 39 sites (*ccb206*) in Arabidopsis [36]. No significant differences could be found in the edition frequency for these 2 genes between WT, KO and OEX plants (data not shown). Thus, Whirlies do not appear to be required for the correct editing process of mtRNA in flowering plants. This is not unexpected since the mechanism of editing in flowering plants is very different than that in *Trypanosoma brucei *(reviewed in [37,38]). In addition, representatives of the Whirly family are found in the green algae *Chlamydomonas reinhardtii *and *Ostreococcus taurii *where the editing process of organellar RNA appears to be absent [39]. The absence of a role for AtWhy2 in the editing process does not preclude the involvement of the Whirlies in other steps of the maturation of RNA.

Finally, although overexpression of *atwhy2 *led to impaired mitochondria, inactivation of this gene in the KO line did not affect plant development and had no detectable effect on mitochondrial morphology and the activity of its respiratory chain complexes. Additionally, no alteration in steady state mtRNA levels or the amount of mtDNA present could be observed. AtWhy2 is thus not essential for the viability of *Arabidopsis thaliana *and the correct function of mitochondria in normal controlled growth conditions. This could be explained by functional redundancy with other proteins present in the organelle. The role of mitochondrial Whirlies could also be highlighted when a particular stress is applied to the plant akin to the transcriptional activator role of StWhy1 which correlates to the induction of its ssDNA-binding activity following biotic stresses. Further experiments will be required on both plastidial and mitochondrial Whirlies before we can actually pinpoint the role(s) of these intriguing proteins in organelles.

## Conclusion

We have shown that AtWhy2 is constitutively bound to mtDNA demonstrating for the first time that Whirlies can associate with DNA in organelles. The interaction between AtWhy2 and mtDNA seems to occur along the entire mitochondrial genome, suggesting that the Whirlies might regulate a variety of processes important for organellar nucleic acid metabolism. Ectopically overproducing AtWhy2 clearly affected the gene expression machinery of mitochondria, reducing both the amount of mtDNA and the level of expression of the genes it encodes. These deficiencies led to smaller plants that exhibited accelerated senescence in leaves. The details in the mechanism of regulation of gene expression by AtWhy2 remain to be unravelled.

## Methods

### T-DNA insertion line characterization

The SALK Institute Genomic Analysis Laboratory provided the sequence-indexed T-DNA insertion line SALK_118907 [40]. These plants contain an insertion at nucleotide +29 relative to the STOP codon. Homozygocity of the insertion was verified by PCR using the following primers: P3, 5'-GTAGCGGCTACTTCATCT CA-3', P4, 5'-TTCGTGTGCACCAAATGCCA-3' and LBb1, 5'-GCGTGGACCGC TTGCTGCAACT-3'. RNA was isolated from homozygous mutants using TRIZOL^® ^reagent (Invitrogen) according to manufacturer's instructions. Absence of expression of AtWhy2 was confirmed by RT-PCR using the following primers: P1, 5'-ATGAA GCAAGCCCGCTCTTT-3' and P2, 5'-CAGCTTTTGTGACAGGAACC-3' and P3 and P4.

### Production of plants constitutively overexpressing AtWhy2 and AtWhy2-myc

Six copies of the c-myc epitope were amplified by PCR from the pCR-blunt-II-TOPO-myc vector (kind gift of Dr. Jeff L. Dangl, University of North Carolina, NC, USA) using the following primers: 5'-CCCAAGCTTGCCCTTCCGGTCGACAA AGCTATG-3' and 5'-CCGCTCGAGTC ATCGATTTCGAACCCGGGGTAC-3'. The amplicon was then digested with HindIII and XhoI and cloned into a pBS-SK(+) vector. The full length *atwhy2 *was amplified from cDNA using the following primers: 5'- ATAAGAATGCGGCCGCATGATGAAGCAAGCCCGCTCTTTG-3' and 5'-GACTAGTTTTATCCCAC TCCAGCTCTAACTG-3'. The amplicon was subsequently digested with NotI and SpeI restriction enzymes and cloned in frame with the c-myc epitopes in the pBS-SK(+)-6-c-myc vector. The tagged *atwhy2 *was then reamplified using the following primers: 5'-TCTAGAGGCGCGCCATGATG AAGCAAGCCCGCTCTTTG-3' and 5'-TCTAGAAGGCCTTCATCGATTTCGA ACCCGGGGTAC-3' cloned into the a pGREENII022935S vector [41]. This construct was cotransformed with companion vector pSOUP into a GV3101 pMP90 *Agrobacterium tumefaciens *strain. Plant transformation was carried out using the floral dip approach as described [42]. Transformed plants were selected on soil using the BASTA resistance conferred by the pGREENII0229 vector. All plants that expressed the construct exhibited the phenotype presented here. Because homozygous lines were almost completely infertile, heterozygous lines were derived from the T1 plants and used for the experiments shown here. WT plants which constitute approximately 1/4 of the progeny were identified by their normal appearance and removed for the experiments involving mature plants. Plants overexpressing an untagged version of *atwhy2 *under the control of a CaMV 35S promoter phenocopied the plants overproducing the AtWhy2-myc construct, thereby eliminating the possibility that the phenotype could be a result of some unspecific effect of the epitope on the biogenesis of mitochondria.

### Antibody production

Recombinant AtWhy2 was expressed and purified as described previously [6]. Rabbits were immunized and antiserum was collected. For protein gel blot analysis, the antiserum was used typically at a concentration of 1:1000 to 1:2500.

### DNA, RNA gel blots and oligonucleotides

RNA was isolated from 4 week old plants using TRIZOL^® ^reagent (Invitrogen) according to manufacturer's instructions. DNA was isolated from plants using a CTAB DNA extraction protocol [43]. Running of the samples and blotting of the gels was performed as described [44]. All PCR products in this study were amplified by PCR from DNA using the following primers: RRNA26SMFOR, 5'-GCGT ACCTTTTGCATGATGGG-3', RRNA26SMREV, 5'-CCTAGCCCATTGAGTGCTCTA-3', NAD7FOR, 5'-TTGCGAGGTACCATTACGA GC -3', NAD7REV, 5'-CACCACTGAATCCCCAATCCT-3', ATP9FOR, 5'-ACCCGAGATGTTAGAAGGTGC-3', ATP9R EV, 5'-GGCCA TCATTGGGGCAAACAA-3', COX1FOR, 5'-GTCTATCCGCCCTTAAGTGGT-3', COX1REV, 5'-TACACCTCTGGATGACCGAAG-3', ATP8FOR, 5'-GGAGATGGAGTACTTG GGATC-3', ATP8REV, 5'-TCCATTCCTCGTGAGCCACTT-3', RPS3FOR, 5'-CCATGA CCGATTACCCTCGATA-3', RPS3REV, 5'-CTGTAAGCTTCTTCCCTGTGC-3', RPL16FOR, 5'-CCTGCGGAAGTATCTACTCGT-3', RPL16REV, 5'-ATGTA GCGGCTTGTCGAGCAT-3', ORF240aFOR, 5'-GCGTTCTAAGATCACTGAGGG-3', ORF240aREV, 5'-AGCTCGTTGAGATCGAGAGCA-3', ATP9ORF262FOR, 5'-GTATAATTCTCAACCCGAGATGTTAGAA-3', ATP9ORF262REV, 5'-GGCTAGATAGCACCATTGTGTCA-3', PSBAFOR, 5'-GGAAGCTGCATCCGTTGATGA-3', PSBAREV, 5'-CCGAATACACCAGCTACACCT-3', UPCOX1FOR, 5'-GCTAGCTCATGGCAGGAAATC-3', UPCOX1REV, 5'-GTAACGTCCGTTCCGTGATCT-3', UPRPS3FOR, 5'-GGGCCCTTTTACCAAGTCTAC-3', UPRPS3REV, 5'-CGCGCTAAGAAAGGTTGCTTC-3', UPRRNA26SFOR, 5'-GCACTCTACCAGAGCTACTAC-3', UPRRNA26SREV, 5'- GGCGCCTTTGTATGTCTCAAC-3', UPRRNA18SFOR, 5'-CCAAGCCTGGATAAGCAACTG-3', UPRRNA18SREV, 5'-CAGTTGCTTATCCAGGCTTGG-3', 5000FOR, 5'- CGATTCATCTCGGCCTTAGAC -3'5000REV, 5'-GTCCTGGAAAGAGCAGATGTG-3', UPATP9652FOR, 5'-GGATCGCATGAGTACGAGAGA-3', UPATP9652REV, 5'-GCACGAGTCGATCCCTATTCT-3', REPEATIIBFOR, 5'-CTAAACGAGACAGCAGCTACC-3', REPEATII REV, 5'-AACAGCCGGAAGGTATGAAGC-3', TRNAGLYFOR, 5'-GTTGGACA TCTGCTCTATCC-3', TRNAGLYREV, 5'-AAGACAGATGCCGCCTACCTA-3', TRNAFMETFOR, 5'-GGTTGTTGGTCCAACGACTCT-3', TRNAFMETREV, 5'-AGTACACACTGTGCACCACGA-3', ER265000FOR, 5'- GTGAATGCCCAGTGCAATCCA-3', ER265000REV, 5'-TTCTCCCTGTGCTTACATGGG-3', ER84000F OR, 5'-TTTCTCGTGCTCTCCGTCCAA-3', ER84000REV, 5'-AGACAGAAGAGCTGGTAGGAC-3', REPEAT1AFOR, 5'-GCCATTCTTCTCTACCCATGC-3', REPEAT1AREV, 5'-CTCACAGAGTCATCGGTATCC-3', REPEAT1BFOR, 5'-TGGACTACCAAAGACCCAGAC-3', REPEAT1BREV, 5'- TCGGATTCTTGCCATCACTGG-3', REPEATIIAFOR, 5'-CGGATCGACTCGACTGATATG-3', REPEATIIAREV, 5'-GTCCTGATCGAGCAACTAGTC-3', NAD3FOR, 5'-TTTACTCCCGATCCGAAGCAC-3', NAD3REV, 5'-TTTGATCCTACTCGGTGTTCC-3', NAD4FOR, 5'-TTCCAAACAGGAACCACCGAT-3', NAD4REV, 5'-CACCAGATTCATATGGGCTAC-3', COX3FOR, 5'-CAAGTCCATGGCCTATTTCGG-3', COX3REV, 5'-ACCATGCAGCTGCTTCAAAGC-3', COX2FOR, 5'-TTGTGATGCAGCGGAACCATG-3', COX2REV, 5'-TGACACCTGAGGAAGGTACAG-3'. All probes were subsequently cloned into a pDRIVE cloning vector (QIAGEN) and sequenced.

### Blue native-polyacrylamide gel electrophoresis (BN-PAGE)

Around 200 mg of leaves were homogenized in 0.33 M Sucrose, 50 mM Bis-tris, pH 7.0 and 750 mM aminocaproic acid. The homogenate was filtered and spun at 1000 g for 10 min. The pellet was discarded and the supernatant spun for 15 min at 10000 g. The pellet enriched with crude mitochondria was resuspended in 50 mM Bis-tris, pH 7.0, 750 mM ACA and 0.5 mM EDTA and solubilised with 1% n-dodecyl-maltoside on ice for 5 min. After centrifugation at 25000 g for 5 min, the supernatant enriched with mitochondrial complexes was supplemented with a 5% (w/v) stock solution of Coomassie blue (Serva) in solubilisation medium to a final ratio of 1:4 (w:w) of Coomassie to detergent, and subjected to BN-PAGE according to [45]. The separating gel consisted of a linear gradient of 5–13% (w/v) acrylamide and a stacking gel of 4.5% (w/v) acrylamide. The cathode buffer used was 50 mM Tricine, 15 mM Bis-Tris pH 7.0 at 4°C and 0.02% Coomassie blue (w/v), freshly prepared and supplemented with 0.02% detergent. Electrophoresis was carried out at 65 V for 1 hour at 4°C or until the proteins entered the stacking gel and then at 140 V, constant voltage, overnight. Following electrophoresis, the gels were rinsed briefly with MilliQ water and then equilibrated in a reaction buffer without reagents for 10 min. The gels were then incubated in fresh buffer plus specific reagents. All steps were carried out at room temperature. The reaction media were as described [16]. The reactions were stopped at various lengths of time by fixing the gels in 45% methanol (v/v) and 10% acetic acid (v/v). The gels were destained overnight in the same solution to remove residual Coomassie blue.

### Semi-quantitative RT-PCR

cDNA was produced from total RNA isolated from 4 week old plants and digested with RNAse-free DNAseI before using RevertAid™ First Strand cDNA Synthesis Kit (Fermentas) according to the manufacturer's instructions. PCR was performed using the following oligonucleotides: NADB18FOR, 5'-ATGGAGGTTCCAGGTTCATCG-3', NADB18REV, 5'-AGGGATAAGAGGAACAGCAGC-3', NAD51FOR, 5'-CACGTCCACGAAGTCCAGATT-3', NAD51REV, 5'-TTCTTGGCTTGCAAAGGGCAG-3', COXVBFOR, 5'-GTGATGGTCATCACCATGTCC-3', COXVBREV, 5'-GGAGGAGAATCGTTTCATCGC-3', COX6BFOR, 5'-TCAAACTCCATCGCTCTCCGA-3', COX6BREV, 5'-AGAGGACCAGGGAATGTTCCA-3', 5At2g45570, 5'-AGCTCTCGCAAGGTCGTTCCATCT-3', 3At2g45570, 5'-CTTCACCTTGTTCTCCCTCAACGT-3', 5At1g47128, 5'-GCAGTTGCTCATCAACCCATTAGC-3', 3At1g47128, 5'-GTCACATTGGGTTGGAGGCTTGAT-3', 5At2g38860, 5'-CGGCGGTTTATGACCTTGAGGATG-3', 3At2g38860, 5'-AAAAAGGCAGGCAGATCCGTGGCT-3', 5-TUBURT, 5'-GGTGGAGCCTTACAACGCTACTTT-3', 3-TUBURT, 5'-TCGCCTGAACATCTCTTGGATCGA-3', 5AT5G45890, 5'-GGCTGCGAAGGCGGTTTAATGGAT-3', 3AT5G45890, 5'-CGCCGTATCCAATCGCAGTTACTG-3'.

### Real-Time Quantitative PCR

DNA extracted from equal fresh weight of each genotype was used as a template. PCR reactions for 384 well plate formats were performed using 2 μl of each DNA sample, 5 μl of the TaqMan PCR Master Mix (Applied Biosystems, CA), 2 μM of each primer and 1 μM of the Universal TaqMan probe in a total volume of 10 μl. Primers for genomic DNA were selected to amplify a unique nuclear region in gene AT5G08530: 5'-GCGTTTGAATCTAGAGAAGGCTA-3' and 5'-CAGAACCAC ATGCATTCTTCC-3'. The Taqman probe #54 from a Universal Library was selected. Primers were selected to amplify a unique mitochondrial region found in the *18S rRNA *gene: 5'-TTTCGAAACCAATTCACTTGAG-3' and 5'-TGTAGATTTC ACCCCTCCACA-3'. The Taqman probe #82 from a Universal Library was selected. Another unique mitochondrial region found in the intron between exons d and e of the *nad5 *gene was also examined using the following primers: 5'-CTCTCCGCAGGG GAATCT-3' and 5'- AACCCCCATGATGTGGTAA-3'. The Taqman probe #38 from a Universal Library was selected. The ABI PRISM^® ^7900 HT Sequence Detection System (Applied Biosystems) was used to detect amplification products and was programmed to an initial step of 10 minutes at 95°C, followed by 45 cycles of 15 seconds at 95°C and 1 minute at 60°C. All reactions were run in triplicate and the average values were used for quantification. The relative quantification of target genes was determined by using the ΔΔCT method. Briefly, the Ct (threshold cycle) values of the mitochondrial region were normalized to the nuclear region (ΔCT = Ct mitochondrial – Ct nuclear) and compared with a calibrator (Col-0 samples): ΔΔCT = ΔCt Sample – ΔCt Col-0 samples. Relative expression (RQ) was calculated using the Sequence Detection System 2.2.2 software (Applied Biosystems) and the formula RQ = 2^-ΔΔCT^.

### Microscopy

For transmission electron microscopy, leaf mesophyll tissue from 4 week old plants was cut into 1–2 mm^2 ^pieces which were washed twice with 0.2 M sodium cacodylate buffer pH 7.4 for 10 minutes. Tissue was fixed by incubation for 6 hours in cacodylate buffer containing 1% EM-grade glutaraldehyde. Samples were coated with epoxy resin, cut into 80 nm sections and mounted onto nickel/formvar grids. Staining of the samples was done using uranyl acetate and lead citrate. Observations were done using a JEOL (JEM1230) transmission electron microscope at 80 kV. Photographs were taken using a Gatan DualVision camera.

### DNA immunoprecipitation in mitochondria

Leaf tissue was fixed in 1% formaldehyde for 15 minutes under vacuum. Glycine was added to 0.125 M to titrate the remaining formaldehyde and fixed tissue was washed 3 times with distilled water. Crude mitochondria were prepared by grinding in mitochondria extraction buffer (350 mM mannitol, 30 mM MOPS pH 7.3, 0.2% bovine serum albumin, 0.6% polyvinylpyrrolidone, 1 mM EDTA) using a mortar and pestle. The ground tissue was filtered through 2 layers of Miracloth (Calbiochem) and spun at 1000 g for 5 minutes in a microcentrifuge to pellet most plastids. The supernatant was recuperated and spun at 11000 g in a microcentrifuge to pellet mitochondria and remaining plastids. The pellet was resuspended in IPP buffer (50 mM Tris-HCl pH 7.5, 150 mM NaCl, 1% NP-40, 0.5% sodium deoxycholate, 1 tablet Complete protease inhibitor (Roche)), lysis was carried out on ice for 2 minutes and the lysate was spun at 11000 g for 15 minutes. Supernatant was recuperated and sonicated using a Branson microtip sonicator. Sonicated material was precleared by incubating with protein-A-agarose beads (Roche) for 1 hour at 4°C on a rotating platform. The precleared material was spun at 11000 g for 15 minutes and the supernatant was transferred to a fresh tube. Proteins were quantified and 100 μg was kept as input material while 500 μg was used for immunoprecipitation using 1/200 rabbit polyclonal anti-AtWhy2 sera in 1 mL final volume. Immunoprecipitations were carried out for 1 hour at 4°C before adding 50 ul of protein-A-agarose beads. Complexes were allowed to form overnight. Beads were pelleted at 100 g for 1 minute and were washed 2 times 5 minutes with IPP buffer, 2 times 5 minutes with low salt buffer (50 mM Tris-HCl pH 7.5, 0.1% NP-40, 0.05% sodium deoxycholate), 2 times 5 minutes with high salt buffer (50 mM Tris-HCl pH 7.5, 500 mM NaCl, 0.1% NP-40, 0.05% sodium deoxycholate), 2 times 5 minutes with LiCl buffer (50 mM Tris-HCl pH 7.5, 0.1% NP-40, 0.05% sodium deoxycholate, 250 mM LiCl) and 2 times 5 minutes with TE pH 7.5 (10 mM Tris-HCl pH 7.5, 1 mM EDTA). Beads were pelleted at 11000 g for 20 seconds and resuspended in TE buffer containing 1% SDS. Complexes were eluted from the beads by heating at 65°C for 15 minutes. Beads were spun down at 11000 g for 5 minutes and the supernatant transferred to a new tube. One tenth volume of 2 M NaCl was added and the eluted complexes were incubated at 65°C overnight for decrosslinking. Glycogen was added to a final concentration of 0.5 μg/ul and twice the volume of 100% ethanol was added. DNA was precipitated at -80°C for 1 hour. Tubes were spun at 11000 g for 20 minutes. DNA pellets were resuspended in TE buffer. DNA was subjected to a phenol/chloroform extraction and to a chloroform extraction before being reprecipitated as above. After a final wash with 70% ethanol, DNA was resuspended in water. This DNA was used for PCR reactions. To further control the specificity of the experiment, we determined the size of DNA fragments following sonication and found that they were between 350 and 1000 bp in length, indicating that for any given region immunoprecipitated specifically, it can be assumed that there is at least one Whirly binding-site within a maximum of 1000 bp starting from the center of the amplified region. Additionally, we used primers to amplify a plastidial DNA region in the *psbA *gene as a negative control since AtWhy2 exclusively localizes to mitochondria and thus should not interact with the chloroplast genome. We found that we could only amplify similar background levels of plastidial genes for Col-0, KO and OEX plants confirming the lack of specific interaction between AtWhy2 and plastidial DNA *in vivo *(data not shown).

## Authors' contributions

AM produced the AtWhy2 and AtWhy2-myc overexpressing lines, characterized the phenotypes of the mitochondrial Whirly mutants, designed the study and drafted the manuscript. JSP obtained the atwhy2 KO plants, helped producing antibodies and performed RT-PCR experiments. MS did the BN-PAGE and in-gel enzymatic assays and participated in the design of the study. FVL participated in the mtDNA immunoprecipitation experiments. CAR participated in the RNA and DNA gel blot analysis of the plants. NB participated in the design and coordination of the study and helped to draft the manuscript. All authors read and approved the final manuscript.
